# Supplementary material for: Differential Expression of miRNAs Between Young-Onset and Late-Onset Indian Colorectal Carcinoma Patients
Source: Noncoding RNA. 2025 Feb 2;11(1):10. doi: 10.3390/ncrna11010010 (PMC11858122; doi:10.3390/ncrna11010010)
Supplement: Supplementary file 1 [file ncrna-11-00010-s001.zip › Supplementary Data Revised.pdf]

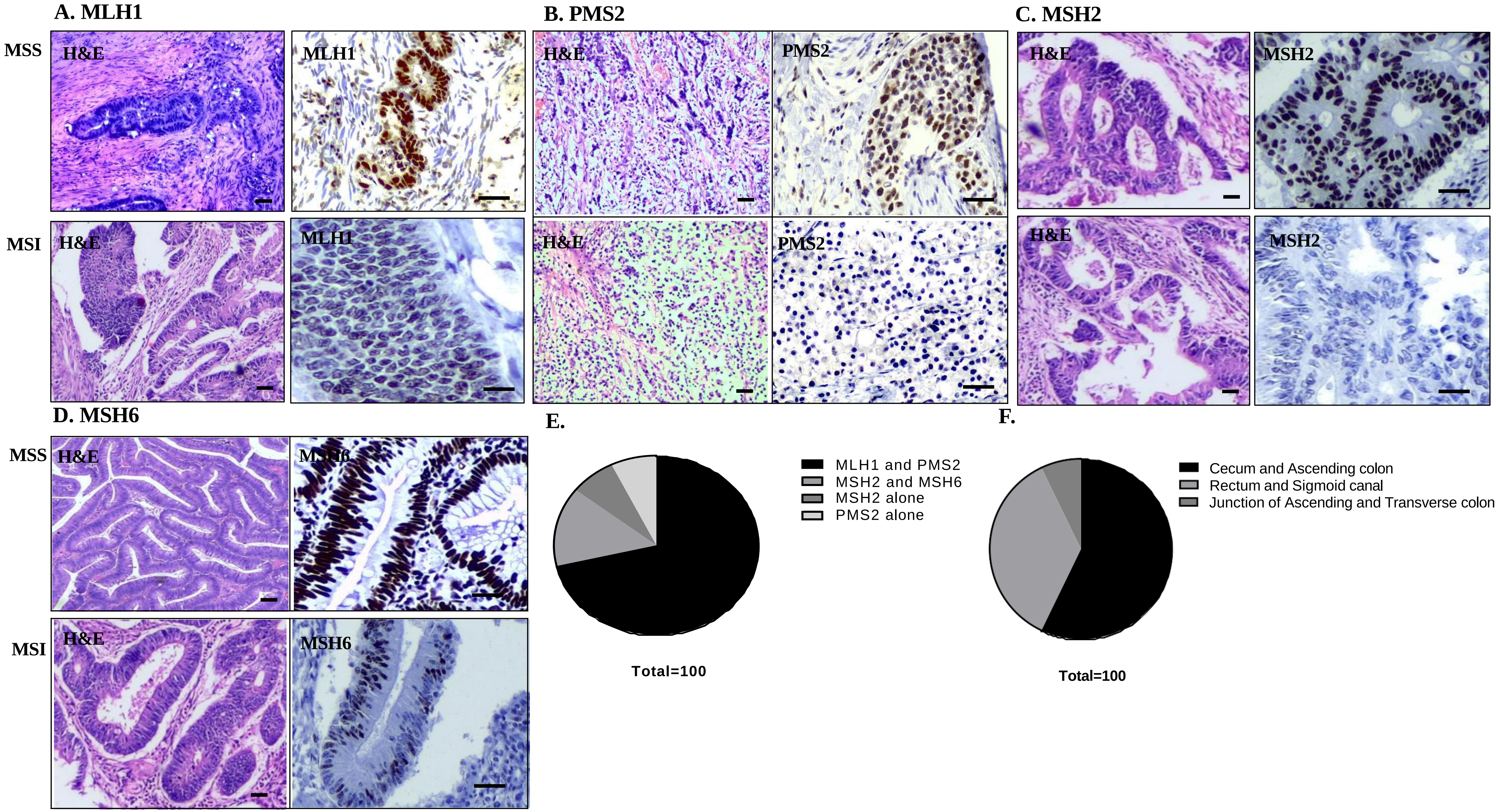

FIGURE S1 IHC based detection of Microsatellite instability markers (MLH1, PMS2, MSH2 and MSH6) in MSI and MSS colorectal tumour tissues

(A – D) Representative micrograph images showing depleted MMR protein in MSI and MSS colorectal tumour tissues. Depicted Haematoxylin & Eosin images are in 10x magnification and IHC images are in 20x magnification. Scale bar represents 100 µm. **E.** Percentage-wise distribution of depleted MMR protein in MSI colorectal tumour tissues. **F.** Percentage-wise distribution of location of dMMR tumours.

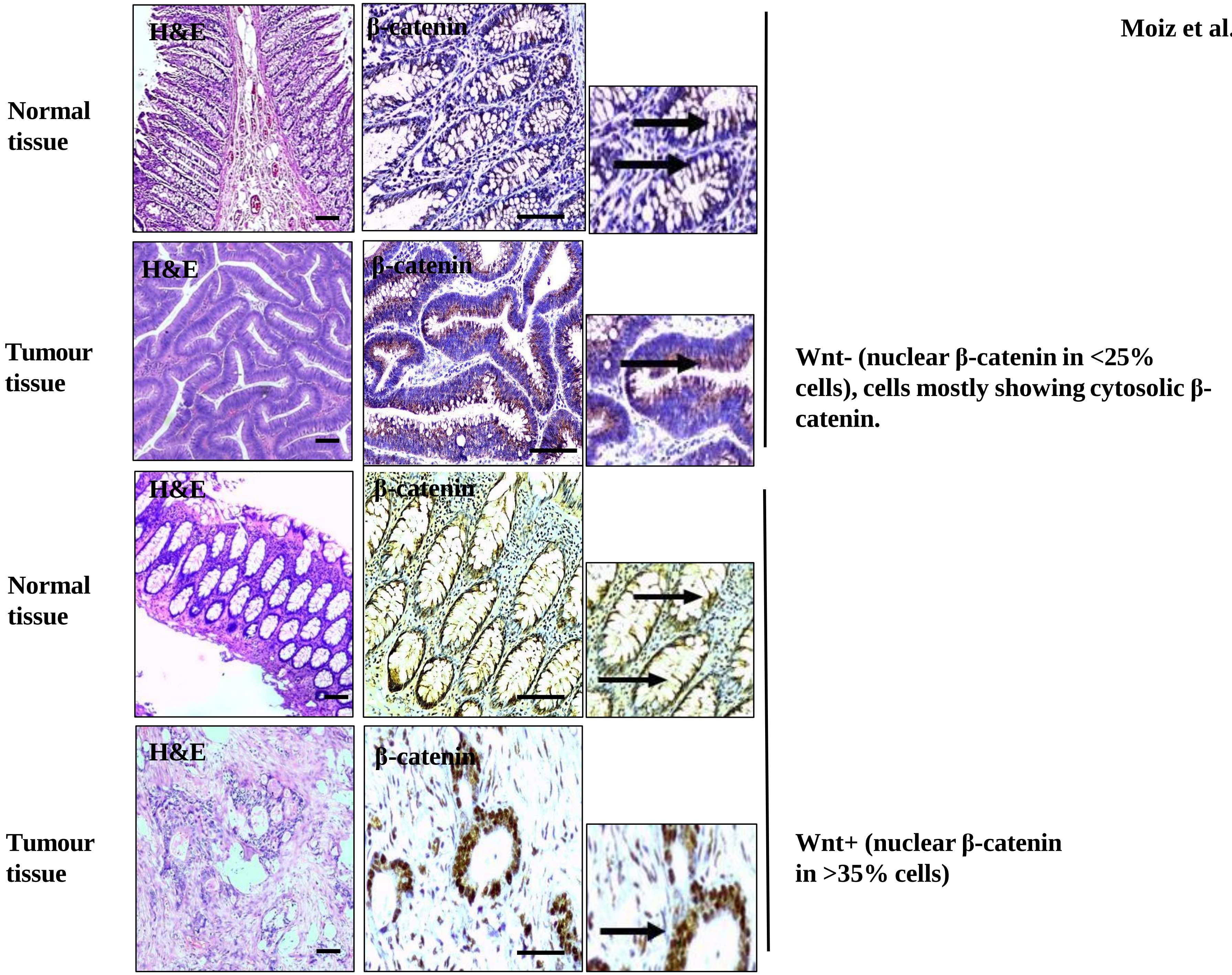

FIGURE S2 IHC based detection of β-catenin in normal and tumour tissues
Representative micrograph images showing IHC based detection of β-catenin in normal and tumour tissues. Normal tissues show membrane localization of β-catenin (inset arrows). Wnt- tumour tissues show mainly cytoplasmic localization of β-catenin (inset arrows). Wnt+ tissues show nuclear β-catenin in >35% cells. Areas marked by black squares have been zoomed into with arrows showing membrane, cytosolic or nuclear localization of β-catenin. Depicted Haematoxylin & Eosin images are in 10x magnification and IHC images are in 20x magnification. Scale bar represents 100 µm.

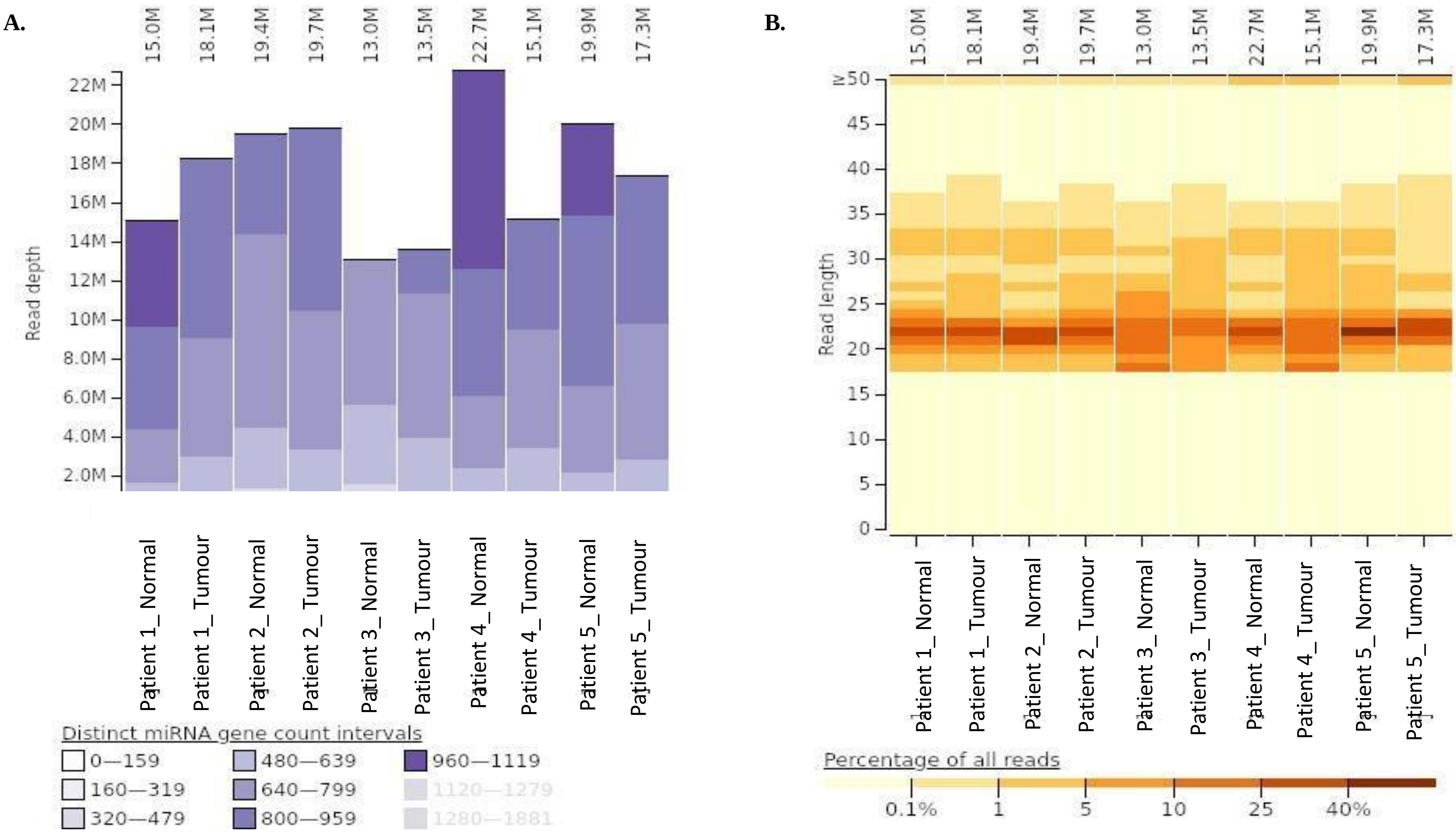

Figure S3 Read Depth Coverage and Sequence Length Distribution Plots

(A) Read depth coverge was estimated using miRTrace v.1.0.1 [59]. Read depth coverage plot showed the read depth (no. Of reads in million) at the y-axis and samples at the x-axis. **(B)** Sequence length distribution was estimated using the miRTrace. Bar diagram was generated using the cleaned sequences of samples. Over 40% sequences in each sample were found to have length distribuion from 18 nt. (minimum trimmed length) to 23 nt.

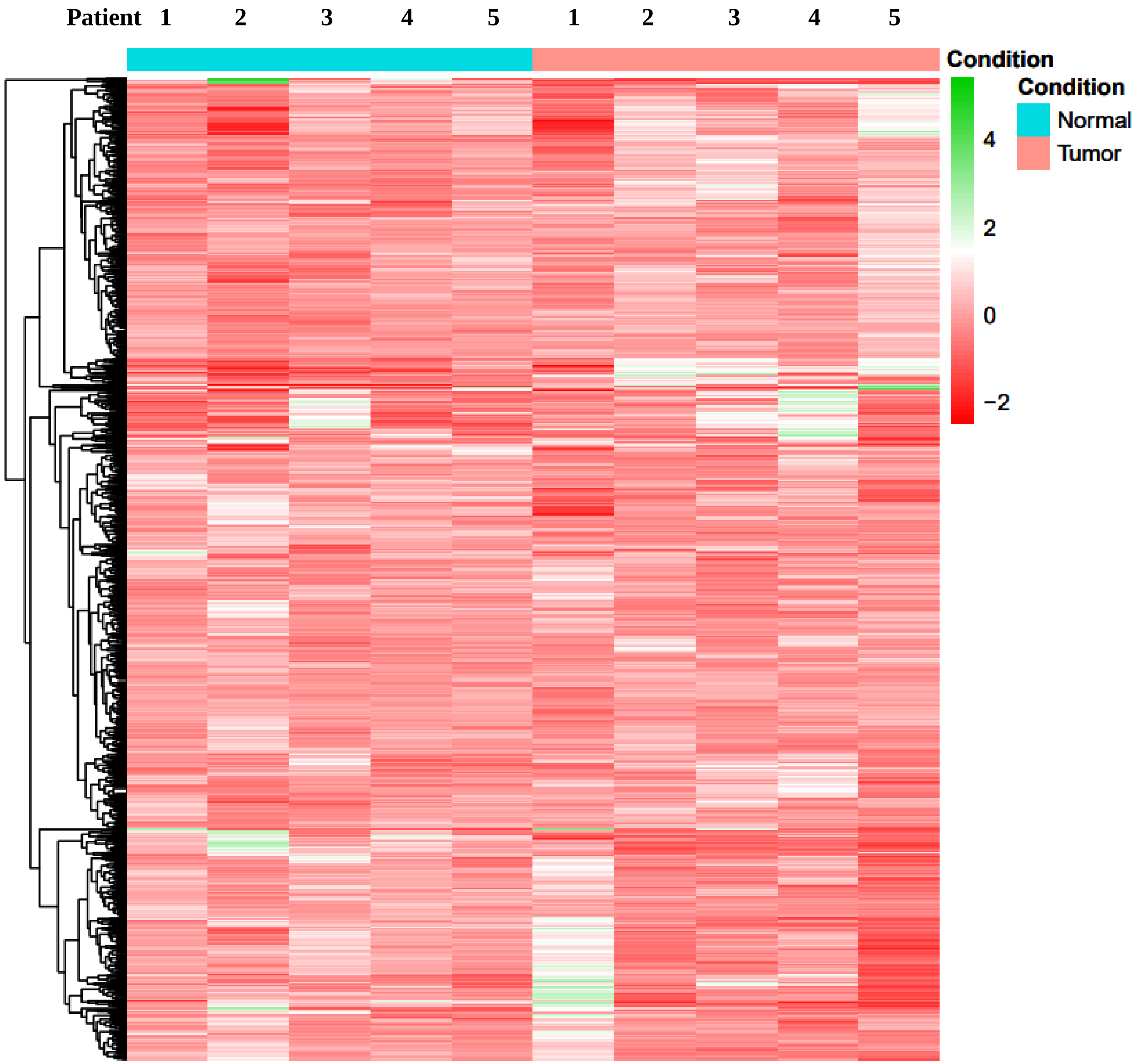

Figure S4 Heatmap of all miRNAs sorted by variance in decreasing order, found across 10 samples (5 normal and 5 tumour)

The heatmap was generated by using rlog-normalized count data of each sample. Z-scores for each miRNA per sample are indicated in red and green colour scale shown on the right, for expression levels below and above mean expressions across sample for that given miRNA, respectively. The condition legend denotes the sample types, where cyan blue is assigned to Normal samples and pink is assigned to Tumor samples.

Supplementary Table 1:

Detailed information on the sequencing miRNA library of CRC tissues: tumor and paired normal tissues.

Sample	Total Reads	Cleared Reads	% Cleared Reads	Total Sequences	Mapped Sequences	% Mapped Sequences
10_normal	15609568	14952600	95.79%	14747826	5359822	36.343%
10_tumour	20282568	18134722	89.41%	17861428	5506910	30.831%
13_normal	20016594	19448545	97.16%	19178794	6863344	35.786%
13_tumour	21324902	19747475	92.60%	19457532	6064884	31.170%
14_normal	15102654	12986956	85.99%	12784955	1038689	8.124%
14_tumour	15628536	13482241	86.27%	13274675	2170200	16.348%
15_normal	23309080	22718559	97.47%	22409421	8376465	37.379%
15_tumour	16669194	15058561	90.34%	14830961	1451003	9.784%
20_normal	20589592	19923833	96.77%	19631591	7282762	37.097%
20_tumour	17753430	17296336	97.43%	17045393	6762325	39.672%

Supplementary Table 2:

List of significant differentially expressed miRNAs identified in multiple sample comparisons of miRNA sequencing results of young (<50 years old) and aged (>55 years old) colorectal cancer apatient tumours (DEM Cut Off: p-value <0.05, Log Fold Change:
>2, <-2)

Comparison (Sample_vs_Control)	miRNA identity	Log_2FC	P-value	Up ↑ / Down↓
All Tumour vs All Normal	hsa-miR-9-5p	-3.08529936	1.73E-05	Down ↓
All Tumour vs All Normal	hsa-miR-129-5p	-3.46090279	3.41E-05	Down ↓
All Tumour vs All Normal	hsa-miR-1-3p	-4.082048456	6.43E-05	Down ↓
All Tumour vs All Normal	hsa-miR-133a- 3p	-4.139735223	0.000118847	Down ↓
All Tumour vs All Normal	hsa-miR-145-5p	-2.927381878	0.000286046	Down↓
Young Tumour vs Young Normal	hsa-miR-1247- 3p	3.70881538	0.00174089	Up↑
Young Tumour vs Young Normal	hsa-miR-27a-5p	2.61304708	0.00901648	Up↑
Young Tumour vs Young Normal	hsa-miR-96-5p	2.80991462	0.02211184	Up↑
Young Tumour vs Young Normal	hsa-miR-148a- 3p	2.49604406	0.02365696	Up↑
Young Tumour vs Young Normal	hsa-miR-135b- 5p	3.41150654	0.02799316	Up↑
Young Tumour vs Young Normal	hsa-miR-378e	-2.61154969	0.00366325	Down↓
Young Tumour vs Young Normal	hsa-miR-326	-2.63728355	0.01712207	Down↓

**Young Tumour vs Young Normal**	**hsa-miR-378c**	**-2.49476989**	**0.01533526**	**Down↓**
**Young Tumour vs Young Normal**	**hsa-miR-378a- 5p**	**-2.48712807**	**0.00620528**	**Down↓**
**Young Tumour vs Young Normal**	**hsa-miR-378d**	**-2.41909916**	**0.01914212**	**Down↓**
**Young Tumour vs Young Normal**	**hsa-miR-363-3p**	**-2.40777269**	**0.04421748**	**Down↓**
**Young Tumour vs Young Normal**	**hsa-miR-143-3p**	**-2.29934116**	**0.01331962**	**Down↓**
**Young Tumour vs Young Normal**	**hsa-miR-504-5p**	**-2.28494574**	**0.01904966**	**Down↓**
**Young Tumour vs Young Normal**	**hsa-miR-139-5p**	**-2.27780576**	**0.0240824**	**Down↓**
**Young Tumour vs Young Normal**	**hsa-miR-139-3p**	**-2.1809868**	**0.00568353**	**Down↓**
**Young Tumour vs Young Normal**	**hsa-miR-9-3p**	**-2.15316894**	**0.00770244**	**Down↓**
**Young Tumour vs Young Normal**	**hsa-miR-490-5p**	**-2.14003278**	**0.01709929**	**Down↓**
**Young Tumour vs Young Normal**	**hsa-miR-133a- 5p**	**-2.13259517**	**0.00324305**	**Down↓**
**Young Tumour vs Young Normal**	**hsa-miR-490-3p**	**-2.11550509**	**0.01130573**	**Down↓**
**Young Tumour vs Young Normal**	**hsa-miR-887-3p**	**-2.10734608**	**0.00627189**	**Down↓**
**Young Tumour vs Young Normal**	**hsa-miR-30a-5p**	**-2.0469516**	**0.00769662**	**Down↓**
**Young Tumour vs Young Normal**	**hsa-miR-133b**	**-2.01434615**	**0.00292072**	**Down↓**

**Young Tumour vs Young Normal**	**hsa-miR-143-5p**	**-2.0070137**	**0.02078392**	**Down↓**
**Old Tumour vs Old Normal**	**hsa-miR-455-3p**	**2.01483885**	**0.008717**	**Up↑**
**Old Tumour vs Old Normal**	**hsa-miR-31-3p**	**3.06621503**	**0.01037511**	**Up↑**
**Old Tumour vs Old Normal**	**hsa-miR-204-5p**	**-4.35495512**	**0.01160546**	**Down↓**
**Old Tumour vs Old Normal**	**hsa-miR-1247- 5p**	**2.63591607**	**0.02448825**	**Up↑**
**Old Tumour vs Old Normal**	**hsa-miR-10524- 5p**	**2.07155472**	**0.02805908**	**Up↑**
**Old Tumour vs Old Normal**	**hsa-miR-549a- 5p**	**3.15057457**	**0.03220999**	**Up↑**
**Old Tumour vs Old Normal**	**hsa-miR-934**	**2.11438475**	**0.03480386**	**Up↑**
**Old Tumour vs Old Normal**	**hsa-miR-135b- 3p**	**2.6736609**	**0.03507436**	**Up↑**
**Old Tumour vs Old Normal**	**hsa-miR-10396b-3p**	**2.00250344**	**0.03772035**	**Up↑**
**Old Tumour vs Old Normal**	**hsa-miR-4485- 3p**	**2.92834074**	**0.04423556**	**Up↑**
**Old Tumour vs Old Normal**	**hsa-miR-31-5p**	**4.86227719**	**0.04948822**	**Up↑**

Supplementary Table 3:

Targets of validated significantly upregulated/ downregulated miRNAs in EOCRC that are differentially expressed in colorectal cancer TCGA datasets (TCGA-COAD and TCGA-READ).

TARGET miR UP/ DOWN	TCGA-COAD and/or TCGA-READ DOWN/UP	SELECTED GENES
Target miR UP<br>Hsa-miR-1247- 3p	TCGA-COAD AND TCGA-READ DOWN	NT5DC3, LIX1L, C3, SSBP2, MDM4, BRSK1, CRISPLD2, KIAA1614, TPCN2, VSTM4, GK5, PHLDA3, PRICKLE1, STX2, ZDBF2, SCARF1, PLEKHG2, PPAP2B, BCL6
	TCGA-READ DOWN ONLY	SEMA3E
Target miR UP<br>Hsa-miR-148a- 3p	TCGA-COAD AND TCGA-READ DOWN	BCL2, RBM38, UNKL, PBXIP1, TNRC6A, RAB12, S1PR1, IGFBP5, STARD13, PRNP, COLEC12, TXNIP, RASSF8, ADARB1, RAB34, GAS1, BMP3, ITGA5, NPTX1, PDK4.
	TCGA-READ DOWN ONLY	GNB5, MTMR9, IL15, EOGT
	TCGA-COAD DOWN ONLY	WNT2B, DNAJB4
Target miR DOWN<br>Hsa-miR-326	TCGA-COAD AND TCGA-READ UP	PKM, PLA2G4F, FASN, KPNA2, ACTG1, NOB1, POFUT1, IHH, CNNM4, CCND1, ABCC6, SF3B3<br>BCL11B, RBM47, WBSCR27, EPHB3, GRWD1, MTHFD2, MOCS3, BMP7, ULBP3, HNRNPA1, ETV4, TFF1
	TCGA-READ UP ONLY	ZNRF2, NKRF, ARL5B, DCAF7, ERBB2, TOMM5, ARRDC1, SPTLC1

Supplementary Table 4: Targets of validated significantly upregulated/ downregulated miRNAs in EOCRC differentially expressed in TCGA-COAD and TCGA-READ and enriched in GO Enrichment pathways.

miRNA	GO Enrichment analysis Category	GO Enrichment Pathway	Pathway Genes
Hsa-miR-1247-3p	Biological Processes	Anatomical structure morphogenesis, Receptor mediated endocytosis, Neuron remodeling, Neuron maturation, Alcohol catabolic proc., Organic hydroxy compound catabolic proc, Cholesterol catabolic proc., Sterol catabolic proc., Positive reg. of axon regeneration, Positive reg. of neuron projection regeneration, Genomic imprinting, DNA methylation involved in embryo development.	BRSK1, SEMA3E, BCL6, C3, VSTM4, CRISPLD2, PRICKLE1, MDM4, STX2, PHLDA3, TPCN2, SCARF1, GK5, ZDBF2.
Hsa-miR-1247-3p	Molecular Function	Low-density lipoprotein particle binding, Phosphatidylinositol-3,5-bisphosphate binding, Lipoprotein particle binding, Protein-lipid complex binding, Scavenger receptor activity,	SCARF1, TPCN2, PHLDA3.
Hsa-miR-148a-3p	Biological Processes	Negative regulation of metabolic processes, Negative regulation of cellular metabolic processes, Regulation of phosphate metabolic processes, Regulation of phosphorus metabolic processes, Tissue morphogenesis, Cellular response to nutrient levels, Negative reg. of anoikis, Regulation of anoikis, Anoikis.	TNRC6A, MTMR9, IGFBP5, RBM38, PBXIP1, BCL2, ADARB1 IL15, PRNP, TXNIP, GAS1, BMP3, PDK4, ITGA5, WNT2B, S1PR1, STARD13.
Hsa-miR-148a-3p	Cellular Component	Ruffle membrane, Leading edge membrane, Ruffle, RISC Complex, RNAi effector complex.	TNRC6A, ITGA5, MTMR9.
Hsa-miR-148a-3p	Molecular Function	Molecular function regulator activity, Chaperone binding, Protein phosphatase binding, Phosphatidylinositol-3-phosphatase activity, Phosphatidylinositol monophosphate phosphatase activity, Phosphatidylinositol phosphate phosphatase activity.	STARD13, WNT2B, BMP3, IL15, GNB5, DNAJB4, BCL2, MTMR9, PRNP, TXNIP.
Hsa-miR-326	Biological Processes	Blood vessel development, Vasculature development, Gland development, Angiogenesis, Response to inorganic substance, Icosanoid transport, Negative regulation of immature T-cell proliferation, Leukotriene transport, Negative regulation of T-cell differentiation in Thymus.	BMP7, POFUT1, ERBB2, CCND1, BCL11B, IHH, FASN, ETV4, ACTG1, EPHB3, PKM, BCL11B, HNRNPA1, ABCC6, TFF1, NOB1, PLA2G4F.
Hsa-miR-326	Cellular Component	Basolateral plasma membrane, Basal plasma membrane, Basal part of cell.	ABCC6, ERBB2, CNNM4.
Hsa-miR-326	Molecular Function	ATPase-coupled inorganic anion transmembrane transporter activity, ABC-type glutathione S-conjugate transporter activity.	ABCC6
